# Visual recovery in a patient with total hyphema, neovascular glaucoma, long-standing retinal detachment and no light perception vision: a case report

**DOI:** 10.1186/1752-1947-5-221

**Published:** 2011-06-17

**Authors:** Olusola Olawoye, Christopher C Teng, Uri Shabto, Jeffrey M Liebmann, Francis A L'Esperance, Robert Ritch

**Affiliations:** 1 Einhorn Clinical Research Center, The New York Eye and Ear Infirmary, New York, NY, USA; 2 Departments of Ophthalmology, New York Medical College, Valhalla, NY, USA; 3 New York University Medical Center, New York, NY, USA; 4 Columbia University College of Physicians and Surgeons, New York, NY, USA

## Abstract

**Introduction:**

We report the case of a patient with total hyphema, neovascular glaucoma, long-standing retinal detachment and no light perception vision, who regained counting fingers vision with complete regression of neovascularization following anterior chamber washout, intravitreal bevacizumab, pars plana vitrectomy, and silicone oil placement. This represents a rare case in which a patient with no light perception vision was able to regain functional vision.

**Case presentation:**

A 63-year-old Caucasian man with a 55-year history of long-standing retinal detachment after trauma presented to our facility with pain and redness, a total hyphema, no light perception vision and an intraocular pressure of 60 mmHg (right eye). He had a history of diabetes mellitus and coronary artery disease. Following anterior chamber washout, he was found to have neovascular glaucoma, for which intravitreal bevacizumab was administered. After washout and intraocular pressure control, his visual acuity improved to light perception. He subsequently underwent vitrectomy, membrane peeling, endolaser and silicone oil placement to reattach his retina, and then a second retinal reattachment procedure. Following these procedures, he had visual recovery to counting fingers vision in his right eye at five metres, complete regression of neovascularization, and intraocular pressure of 10 to 12 mmHg on one antiglaucoma medication.

**Conclusion:**

Functional vision can be regained despite long-standing retinal detachment.

## Introduction

Long-standing retinal detachments (over one year) with poor visual acuity are typically associated with cystic degeneration of the macula and retina, loss of pigment from the underlying retinal pigment epithelium, proliferative vitreoretinopathy, and poor visual outcome after retinal reattachment surgery [1].

Chronic retinal detachment is a cause of rubeosis iridis and neovascularization of the anterior chamber angle with subsequent neovascular glaucoma (NVG). NVG represents one of the most severe forms of secondary glaucoma, caused by a number of ocular and systemic conditions. Retinal ischemia and hypoxia initiate the release of angiogenesis factors, with consequent development of new vessels.

We report the case of a patient with total hyphema, NVG, long-standing retinal detachment and no light perception (NLP) vision, who regained counting fingers (CF) vision with complete regression of the neovascularization following anterior chamber (AC) washout, intravitreal bevacizumab, and two retinal reattachment surgeries.

## Case presentation

A 63-year-old Caucasian man presented to our facility with a four-week history of pain and redness in his right eye. He had had a traumatic retinal detachment of the right eye (55 years ago) after being struck in the eye with a stone. He subsequently developed a cataract in his right eye, for which he underwent cosmetic lensectomy at age 25. His best corrected visual acuity post lensectomy was light perception (LP), with a persistent retinal detachment. He was left aphakic in his right eye. At age 39, he had laser retinopexy in his left eye for lattice degeneration. He had a history of diabetes mellitus, quadruple cardiac bypass surgery, and defribillator implantation.

**Figure 1:**
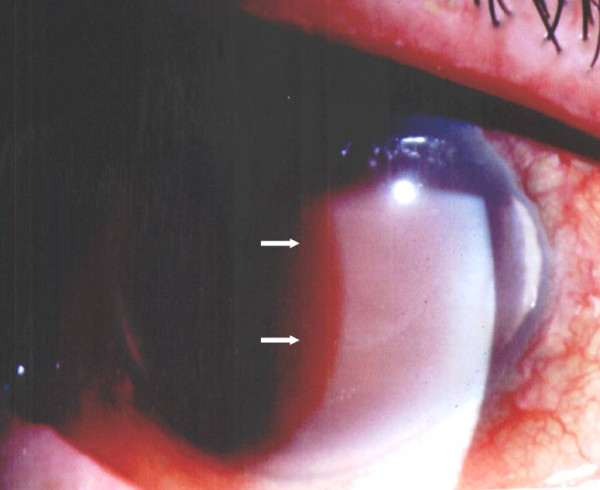
**Total hyphema (right eye)**.

**Figure 2:**
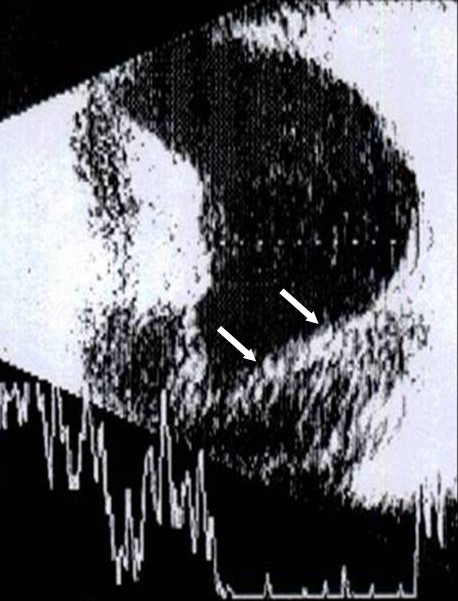
**Ultrasonography (right eye) at presentation, indicating low retinal detachment and vitreous hemorrhage**.

On examination, his visual acuity was NLP (right eye) and 20/20 (left eye). External examination showed ptosis and exotropia in his right eye. Slit lamp examination revealed right eye nasal and temporal band keratopathy, mild corneal edema, total hyphema and no posterior view given the hyphema (Figure 1). He had an unremarkable examination of his left eye, with early nuclear sclerosis. Intraocular pressure (IOP) by Goldmann applanation tonometry was 60 mmHg (right eye) and 10 mmHg (left eye). Dilated fundus examination of his left eye revealed two areas of laser retinopexy surrounding lattice degeneration at 1:00 and 3:00 o'clock. Ultrasound of the right eye revealed low-lying retinal detachment with vitreous hemorrhage (Figure 2).

Immediate AC paracentesis to relieve pain and pressure reduced the IOP to 38 mmHg. Over the following two weeks, his IOP fluctuated between 36 to 50 mmHg (right eye), with no resolution of the hyphema or pain. AC washout was performed, and during surgery he was noted to have NVG with 360° rubeosis iridis, and vitreous hemorrhage with ghost cells. Over the next three weeks, he had two doses of 1.25 mg/0.05 ml intravitreal bevacizumab two weeks apart to treat his neovascularization.

Over eight weeks, his IOP gradually decreased to 15 mmHg (right eye) on four antiglaucoma medications, and his visual acuity improved from NLP to LP. His retina was noted to be normal in color and not necrotic or cystic. Given the good appearance of the retina and because he had recovered LP vision, we decided to see if vision would improve further by repairing the detachment. At two months after AC washout and three months after presentation, pars plana vitrectomy, membrane peel, retinotomy with aspiration of subretinal blood, endolaser retinopexy, inferior iridotomy, air/fluid exchange and retinal reattachment with silicone oil were performed.

**Figure 3:**
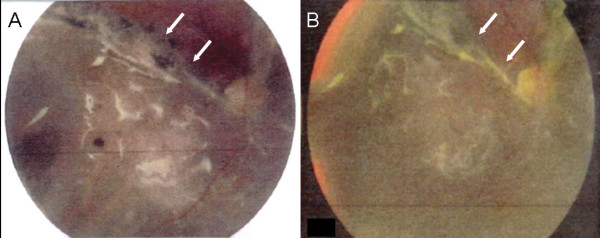
**The right eye following first retinal detachment repair (A) and second retinal detachment repair (B)**.

Following surgery, his vision improved to counting fingers vision in the right eye at five metres, with IOP of 12 to 17 mmHg (right eye) on two antiglaucoma medications. There was complete regression of the rubeosis. His IOP remained stable over the next year on the same medication regimen. Fundus photography performed during a follow-up visit revealed a flat retina in both eyes, though there was residual fibrosis in the right eye (Figure 3). However, one year after retinal reattachment, he was noted to have an inferior tractional retinal detachment in the right eye with areas of subretinal fibrosis.

He subsequently had a second membrane peeling, removal of subretinal membranes, drainage of subretinal fluid, controlled retinectomy, and endolaser retinopexy. Postoperatively, his best corrected visual acuity remained CF in the right eye at five metres and his IOP remained stable at 10 to 12 mmHg on timolol 0.25% once daily. The optic nerve and macula had retinal pigment epithelial hypertrophy and subretinal fibrosis (Figure 3). Our patient is currently being monitored with visual acuity of CF at five metres in the right eye and IOP 19 mmHg on timolol 0.25% once daily at last follow-up.

## Discussion

With modern surgical techniques, a greater than 90% primary anatomic success rate can be expected following retinal detachment repair [1]. Despite this high level of anatomic success, visual results may remain compromised because of permanent functional damage due to macular detachment. The most important predictor of visual recovery after retinal detachment repair is pre-operative visual acuity, which is directly related to the height of macular detachment [2]. A shorter duration of detachment and younger age are also important in visual recovery. Visual recovery following macula-off retinal detachment declines in an exponential fashion in relation to increasing duration of the detachment [3].

Chronic retinal detachments can also lead to complications such as proliferative vitreoretinopathy and rubeosis iridis. Iris neovascularization (INV) and NVG are highly correlated with retinal ischemia, which stimulates production of vascular endothelial growth factor (VEGF), a key molecule mediating neovascularization [4]. Intravitreal injection of VEGF has been shown to produce INV and NVG in non-human primates, and inhibition of endogenous VEGF is effective for suppressing the retinal ischemia induced INV [5].

Bevacizumab (Genentech, San Francisco, CA, USA) is a full-length humanized monoclonal antibody that binds all isoforms of VEGF. Recent reports using intravitreal bevacizumab injections have reported rapid and marked regression of neovascular vessels in INV and NVG [6]. Complete resolution of iris and angle neovascularization has also occurred after intravitreal bevacizumab.

Our patient presented with a total hyphema, NVG, elevated IOP, and long-standing traumatic retinal detachment with NLP vision. Trauma accounts for approximately one in 10 retinal detachments; the visual prognosis for eyes with NLP vision after trauma is dismal [7]. Of 52 eyes with a presenting vision of NLP, two improved to hand motion and two improved to LP vision following surgery [7]. Eyes with an initial acuity of hand motions or better correlated with significantly better visual outcome, but when the initial vision was LP or NLP, poor visual outcomes (57% to 100%) were more likely.

Brinton *et al*. [8] reported a series of 106 eyes with trauma involving the posterior segment; 55 eyes (52%) achieved final visual acuity of 20/100 or better following surgery. The eyes that underwent vitrectomy within 14 days of the injury had a better final visual outcome than those that underwent later vitrectomy. In 1982, Burton [3] reported that of patients with macula-off retinal detachments, 53% of patients who underwent surgery by nine days achieved visual acuity 20/20 to 20/50, with poor outcomes for long-standing detachments.

Despite our patient's 55-year duration of long-standing retinal detachment, following AC hyphema washout, the retina had good color. Given this finding, the decision was made to repair the detachment and he was able to regain CF vision after two retinal surgeries. Suzuki and Hirose [9] reported a case of visual recovery from NLP in total retinal detachment of three months duration. Their patient was able to regain CF vision after two surgeries and postulated that some retinal receptors were able to escape deterioration.

We believe that our patient was able to regain vision because of the low height of the long-standing retinal detachment. Previous studies have shown a positive relationship between the extent of the macular elevation and final visual acuity [3]. In experimental detachments in owl monkeys, Machemer [10] found that photoreceptor cell degeneration increased as the distance between the pigment epithelial layer and the photoreceptors increased. Our patient likely had areas of neurosensory retina intact, which allowed him to have some visual recovery after the retinal procedures.

Additionally, IOP control likely contributed to the improvement of vision. Wittstrom *et al*. [11] reported that a significant lowering of IOP seemed to improve the function of the central retina, as demonstrated by increased amplitudes and reduced implicit times assessed with multi-focal electroretinography.

To the best of our knowledge, there has been no previous similar report of visual recovery in a patient with long-standing traumatic retinal detachment. We hope that with future advances, stem cells and retinal progenitor cells may be transplanted into diseased retinas to integrate and develop synaptic connections with host cells, and further improve visual function.

## Conclusion

Functional visual recovery is possible despite long-standing retinal detachment with NLP vision.

## Consent

Written informed consent was obtained from the patient for publication of this case report and any accompanying images. A copy of the written consent is available for review by the Editor-in-Chief of this journal.

## Competing interests

The authors declare that they have no competing interests.

## Authors' contributions

OO and CCT were involved in acquiring data, conception, design and writing the manuscript; US and FAL were involved in patient care and manuscript preparation; RR and JML were involved in patient care, conception, design, drafting and revising the manuscript. All authors have read and approved the final manuscript.
